# A qualitative study exploring healthcare providers’ and trainees’ barriers to COVID-19 and influenza vaccine uptake

**DOI:** 10.1080/21642850.2022.2106231

**Published:** 2022-08-04

**Authors:** Abhinand Thaivalappil, Ian Young, Melissa MacKay, David L. Pearl, Andrew Papadopoulos

**Affiliations:** a Department of Population Medicine, University of Guelph, Guelph, Canada; b School of Occupational and Public Health, Ryerson University, Toronto, Canada

**Keywords:** Vaccine, COVID-19, influenza, barriers, health psychology

## Abstract

**Background:**

Vaccines are effective biological interventions which reduce health burdens and protect healthcare providers from vaccine-preventable diseases. However, there are concerns about varying levels of vaccination coverage of influenza and COVID-19 vaccines among those working in healthcare. The aim of this study was to identify barriers and facilitators to COVID-19 and influenza vaccinations among healthcare providers and trainees using the Theoretical Domains Framework (TDF).

**Methods:**

Semi-structured interviews (*n* = 18) were carried out with healthcare providers and trainees in Canada. A thematic analysis approach was used to code interview transcripts and match findings to TDF domains and broader categories.

**Results:**

Three overarching themes were generated from six TDF domains and three inductively generated categories: (1) making informed health decisions with an added responsibility to protect oneself and patients; (2) a pro-vaccine social network, widespread accessibility, and pursuing a sense of normalcy; and (3) seeking a more nuanced, respectful, and calculated approach to vaccine communication and policy implementation.

**Conclusion:**

These findings help to identify factors associated with influenza and COVID-19 vaccine uptake among individuals in the healthcare field. Addressing these factors may improve healthcare provider sentiments surrounding vaccines, lead to better patient education, and increased uptake of vaccinations with the potential for seasonal booster doses.

## 1. Introduction

Vaccines are one of the most effective interventions in reducing mortality, improving health outcomes, and reducing economic burdens associated with infectious diseases (Andre et al., 2008; Rodrigues & Plotkin, 2020). They protect healthcare providers (HCPs) from public and occupational exposure to vaccine-preventable diseases, reduce susceptibility to hospital-acquired infection, lower disease severity, support healthcare infrastructure, and protect the patients they serve (Lauring et al., 2022; Maltezou et al., 2019). However, evidence suggests providers may adopt misconceptions about vaccines, and some are reluctant or delay receiving COVID-19 and influenza vaccines despite recommendations and accessibility through their workplaces (Dubé et al., 2013; Hollmeyer et al., 2009; Sallam, 2021; Shekhar et al., 2021). A systematic review of global COVID-19 vaccine intentions during the COVID-19 pandemic revealed varying levels of self-reported intentions among nurses (40–63%), healthcare workers (28–77%), and doctors and trainees (62–78%) (Sallam, 2021). Although intentions were poorer, studies conducted later during the pandemic revealed high COVID-19 vaccine uptake (Green-Mckenzie et al., 2021; Kim et al., 2021), suggesting health campaigns, organizational policies, and vaccine mandates were effective at increasing coverage (Schumacher et al., 2021). Despite this intended positive outcome, some HCPs report concerns about COVID-19 vaccines (Kim et al., 2021), and many studies point to low influenza vaccine coverage among providers (Dubé et al., 2014; Genovese et al., 2019; Lam et al., 2010; To et al., 2016) indicating vaccine campaigns and interventions can be further improved. This hesitancy indicates potential gaps in the application of behavioural interventions and current vaccine-related communication.

Vaccine hesitancy is defined as a refusal or delay in the acceptance of vaccines when vaccination services and recommended vaccination schedules are available (Dubé et al., 2013). Overcoming hesitancy within HCPs is a significant public and occupational health concern particularly because providers are often the preferred source of information for patients discussing vaccination, and widespread vaccine acceptance among providers may enhance vaccination coverage within the public (Dubé et al., 2013; Latkin et al., 2021; Maltezou et al., 2019; Ogilvie et al., 2021). A review of studies on uptake of influenza vaccines found healthcare workers primarily get vaccinated for their own benefit (vs. protecting patients) (Dubé et al., 2014). Moreover, HCPs’ knowledge and attitudes are strong determinants of vaccine acceptance and predict intentions to recommend vaccines to patients (Clark, Cowan, & Wortley, 2009; Zimmerman et al., 1997). However, most behavioural science and health communication research on COVID-19 vaccines among HCPs is quantitative (Green-Mckenzie et al., 2021; Kim et al., 2021). Some researchers have investigated both COVID-19 and influenza vaccines as a topic of interest, and these have also been quantitative (Grochowska et al., 2021; Kwok et al., 2021) and focus on vaccination in the context of the COVID-19 pandemic (Di Giuseppe et al., 2021; Gagneux-Brunon et al., 2021; Silva, Bratberg, & Lemay, 2021). To our knowledge, no such study to date has used qualitative methods to compare attitudes and behaviours concerning COVID-19 and influenza vaccines among HCPs. Qualitative methods complement quantitative research by answering questions which quantitative approaches cannot reach, contributing to our understanding of different perspectives in healthcare, and generate data on beliefs and behaviour which can guide policy actions (Green & Thorogood, 2018; Pope & Mays, 1995). There is need for qualitative research to explore potential factors driving COVID-19 and influenza vaccine acceptance among providers who are susceptible to exposure and transmission of these viruses.

The purpose of this study was to conduct semi-structured interviews with Canadian HCPs of different professions to identify which barriers and facilitators influence COVID-19 and influenza vaccine uptake among this group, offer a cross-comparison between how providers perceive influenza and COVID-19 vaccines, and outline recommendations for effective health communication to HCPs. Vaccine uptake is a vaccine-, context – and profession-specific issue (Maltezou et al., 2019), and this research aims to contribute to the current available evidence by sharing detailed insights on enhancing vaccine acceptance and uptake within this population.

## 2. Methods

### 2.1. Study design

This research used a qualitative description design with one-on-one semi-structured interviews to understand the worldviews and perspectives of HCPs and trainees (Bradshaw, Atkinson, & Doody, 2017). All authors come from a public health or epidemiology background and have been involved in disease prevention and health promotion initiatives and research. Two co-authors have expertise in health behaviours, and four have been involved in COVID-19 vaccine initiatives and health communication. The reporting guidelines under the Standards for Reporting Qualitative Research were followed (O’Brien et al., 2014).

### 2.2. Framework

The Theoretical Domains Framework (TDF) aims to understand the context of behaviour and was selected to allow this research to more easily be translated into practice. The TDF was originally formed from 128 constructs and 33 behaviour change theories and synthesized into 14 theoretical domains and informs interventions to bring about behaviour change (Cane, O’Connor, & Michie, 2012). The framework has been applied to understand barriers and facilitators for various implementation problems and health-related behaviours (Cane et al., 2012; Griffiths, Naughton, & Brown, 2021; Kirk et al., 2016). Previous research investigating vaccine hesitancy and uptake have successfully used the TDF to guide their research exploring vaccine hesitancy and uptake (Gallant et al., 2021; Griffith, Marani, & Monkman, 2021; Williams et al., 2020). To our knowledge, this is the first study of its kind to apply it to the HCP population and exploring multiple vaccines.

### 2.3. Ethics

Ethical approval was granted by the Research Ethics Board at the University of Guelph (REB#21-10-024). An electronic copy of the consent form was shared with participants at least two days prior to the scheduled interview date and discussed by the interviewer at the beginning of the interview with all participants.

### 2.4. Participants

Participants were recruited across two groups: HCPs and trainees (i.e. medical students, nursing students). For the sake of this study, we use Statistics Canada’s definition for a HCP which is ‘a health professional that a person sees or talks to when they need care or advice about their health’ (Statistics Canada, 2019). Individuals were required to meet the following inclusion criteria to participate: be an active or retired HCP or a student in a healthcare field, a resident of Canada, able to give informed consent, 18 years old or older, and English-speaking. All participants were compensated with a $20 e-transfer for their time.

Purposive and snowball sampling approaches were used for this study. Recruitment was achieved using 3 strategies: (a) social media (i.e. LinkedIn, Facebook, Twitter) posts via the Canadian Public Health Association (CPHA) networks, (b) CPHA’s e-newsletter, and (c) reaching out to AT’s personal contacts and sharing the recruitment poster within their networks. Data collection continued until no further individuals contacted the research team for interest in participating in the study. Data saturation was obtained in some professions and not among others, and this is discussed in our limitations.

### 2.5. Procedure

One-on-one semi-structured interviews were conducted via telephone between February–March 2022. Interviews were conducted following the approval of the third COVID-19 vaccine dose for adults across Canada in December 2021 (Public Health Agency of Canada, 2022). The interview guide provided in the Appendix was adapted from a previously published study which successfully applied the TDF in qualitative research (Kirk et al., 2016), and questions were reframed to fit the topics of interest in our study. Additional factors of interest were included such as cultural norms, trust in government, and reputation of vaccines based on conversations with HCPs and public health experts as being potentially relevant in one’s decision to be vaccinated.

We explored trainees’ and HCPs’ attitudes, beliefs, and perspectives toward COVID-19 and influenza vaccine uptake. Both vaccines were included in our investigation to compare perceived similarities and differences to barriers of receiving a vaccine. The interview guide consisted of 18 questions (e.g. How familiar are you with COVID-19 and influenza vaccines? Does the accessibility of a vaccine impact you in wanting to get or not to get vaccinated?) and focused on the domains outlined in the TDF. No demographic factors were collected from participants besides gender identity and primary profession. All questions were reviewed by members of the research team and pilot tested by five individuals. This process resulted in questions being rephrased, deleted, added, and merged. Interviews lasted approximately 30 min on average and ranged between 16–56 min.

### 2.6. Analysis

All interviews were audio recorded, transcribed verbatim, deidentified, and participants were assigned pseudonyms. Thematic analysis was conducted from a contextual constructionism epistemological position (Braun & Clarke, 2006; Madill, Jordan, & Shirley, 2000). We employed a semantic approach by matching findings to TDF domains and taking a more deductive approach to coding. A list of all codes and their descriptions are provided in the Appendix. Underlying belief statements were identified, and overarching themes were generated from these codes. Some domains did not fit any themes, especially when participants showed no evidence of specific beliefs influencing vaccine uptake. Participants’ audio recordings were revisited by AT to ensure themes were supported by the data and to consider inflections, emphasis on words, emotions, and pauses.

Triangulation was also employed. Peer debriefing and discussions between researchers were conducted throughout the analysis to generate richer interpretations of data, promote reflexivity, and enhance collaboration. Furthermore, member checking was conducted to increase trustworthiness where participants were invited to provide feedback on the main findings.

Analysis was conducted using NVivo Release 1.6.1 qualitative analysis software (QSR International, Doncaster, Australia). All steps were completed by AT and reviewed by MM and IY. Sub-themes and themes were revised through discussion between the research team.

## 3. Results

Eighteen HCPs and trainees participated in semi-structured interviews. All participants interviewed were vaccinated against COVID-19. Only one individual did not receive their COVID-19 vaccine booster (i.e. third dose) or their influenza vaccination (Table 1). During these interviews, most participants expressed favourable intentions toward the COVID-19 and influenza vaccines. Some participants’ intentions to get the COVID-19 vaccine did not change even if they had a COVID-19 infection during the pandemic. Participant characteristics are presented in Table 1.

**Table 1.** Participant characteristics and immunization status of healthcare providers and trainees who participated in one-on-one semi-structured interviews exploring barriers to vaccine uptake (*n* = 18).

Pseudonym	Gender	Primary profession	Immunization	
			Influenza	COVID-19
Alejandra	Female	Medical Student	Yes	Yes; 3 doses
Claire	Female	Registered Nurse	Yes	Yes; 3 doses
Elisabeth	Female	Pharmacy Technician	Yes	Yes; 3 doses
Ellen	Female	Pharmacy Manager	Yes	Yes; 3 doses
Jade	Female	Medical Student	Yes	Yes; 3 doses
Jilo	Female	Nursing Student; Unit Clerk	Yes	Yes; 3 doses
Julian	Male	Medical Student	Yes	Yes; 3 doses
Katherine	Female	Nursing Student	Yes	Yes; 3 doses
Lindsay	Female	Undisclosed	Yes	Yes; 3 doses
Madeline	Female	Registered Nurse	Yes	Yes; 3 doses
Noelle	Female	Registered Dietitian	Yes	Yes; 3 doses
Rico	Male	Medical Student	Yes	Yes; 3 doses
Sayyid	Male	Medical Student	Yes	Yes; 3 doses
Selena	Female	Medical Student	Yes	Yes; 3 doses
Tony	Male	Medical Student	Yes	Yes; 3 doses
Victoria	Female	Registered Nurse	Yes	Yes; 3 doses
Wendy	Female	Medical Student	Yes	Yes; 3 doses
Xue	Female	Registered Nurse	No	Yes; 2 doses

During thematic analysis, several predominant domains from the TDF were identified to be important factors which influenced vaccine uptake. These included: (1) knowledge, (2) environmental context and resources, (3) social influences, (4) beliefs about consequences, (5) professional role and identity, and (6) emotion. Three additional constructs were found to be salient and recurring: (1) government and public health, (2) cultural and societal factors, and (3) skepticism of pharmaceutical companies. Sub-themes and themes were generated from the data. We describe findings from the thematic analysis below. Additional illustrative quotes under each finding are provided in the Appendix.

### 3.1. Theme 1: making informed health decisions with an added responsibility to protect oneself and patients

Four domains were grouped under this theme: knowledge, professional role and identity, beliefs about consequences, and emotions. Most providers felt they had adequate knowledge on vaccines but indicated a desire to learn more about the COVID-19 vaccines and mRNA technology. Government sources, peer-reviewed literature, and verified organizations and individuals on Twitter were used as the primary sources of information on vaccines and to stay up to date. Misinformation and the lack of long-term data was often discussed without prompts:

Tony: The information that isn't available, something that just might just not be known at the moment, for example, [when] can we talk about long-term effects? Maybe 20 years from now? It's just something that we don't, we can't say with certainty, right? But we can make a very educated guess on it based on previous models of the vaccine.

Furthermore, almost every participant felt it was their responsibility to get vaccinated and stay informed, but some voiced it should be of their own volition rather than through mandates:

Alejandra: … people do look for healthcare workers as sources of knowledge. [It is] a responsibility for me to have that information if people were turning to me and asking these questions.

Participants had a desire to protect themselves and others. Risk perceptions about influenza were slightly lower compared to COVID-19. Some reported not seeing any cases of influenza in recent years which led them to not having strong intentions to receiving the vaccine. Others received the influenza vaccine mainly to protect others and because it was required by their school or workplace:

Victoria: As a nurse working with vulnerable people, working in the health care system, it is our responsibility to get vaccinated as well because we know there's a higher risk of us spreading COVID for unvaccinated. So, if I'm working with patients, I want to be able to protect them and I firstly protect myself if they have a COVID infection.

Most HCPs and trainees stated that emotions did not play a role in their decision to vaccinate, but some alluded to positive and negative feelings even if emotions did not impact their decision to receive the COVID-19 vaccine:

Lindsay: … having that peace of mind with having some sort of immunity and not being as fearful to do regular, almost regular things, without fear of catching it.

### 3.2. Theme 2: a pro-vaccine social network, widespread accessibility, and pursuing a sense of normalcy

Three constructs were grouped under this theme: social influences; societal and cultural norms; and environment, organizational context, and resources. Most participants had a pro-vaccine social circle but knew people in their extended network who were not vaccinated. Some participants were a source of information for friends and family members who were not in healthcare, or they discussed the pandemic and vaccines with their peers which may have reinforced their decision to vaccinate. None discussed influenza vaccines in this context:

Ellen: My sister-in-law was pregnant, so she at one point was texting me being like, ‘*Do you think I should get this now?’* because she was a little bit worried. I've had some friends ask about it as well, like in terms of ‘*What should I get?’* Those types of questions.

Jade: … influenza isn't as prevalent or at least not in our minds right now.

COVID-19 vaccines were associated with a sense of normalcy in the community, lifting restrictions, and signifying the end of the pandemic. Some individuals felt society at large was targeting unvaccinated and hesitant individuals or taking away individual rights. Most participants cited accessibility as the largest facilitator in getting vaccinated:

Jade: I think health care providers are in an incredibly privileged position where vaccine availability or vaccines are quite available to us … Actually, the flu shot I had quite a bit of trouble getting. It was, yeah, I was looking at different pharmacies and there were a lot of pharmacies that didn't have any doses available. In the end, I was able to get it through the hospital, which was, you know, another kind of privilege as a healthcare worker.

The only profession-specific barriers toward uptake observed were under this finding. Participants felt there were inadequate resources in some areas such as personnel in occupational settings (e.g. pharmacy), overcoming language barriers for cultural groups, accessibility for seniors as many of the resources were online or virtual, combating misinformation, and curbing anti-vaccine sentiments:

Jade: This time around with the COVID vaccine, I did see a lot of unequal access, whether it be not having access to a device that connects to the internet that you can book an appointment on (inaudible). This time with COVID, we were lining up and hunting for vaccines, right? So as simple as not being able to have an hour in the middle of the day to go line up for a vaccine. I think these are issues a lot of patients face.

### 3.3. Theme 3: seeking a more nuanced, respectful, and calculated approach to vaccine communication and policy implementation

Two constructs were grouped under this theme: skepticism of pharmaceutical companies; and government and public health. Participants questioned the motives behind pharmaceutical companies pushing for COVID-19 vaccinations despite most having a strong awareness of the health benefits these vaccines offered. A few others were apprehensive or even critical about the third COVID-19 vaccine dose. They suggested boosters could be administered strategically to certain at-risk groups:

Rico: I totally believe the boosters are beneficial in terms of a health perspective. But at the same time, I did start to question to myself wondering whether or not there was a potential sort of money grab associated with this, and I think that maybe some information is left out to the public.

The vaccine brand or company (e.g. Pfizer, Moderna) was not a factor in one’s decision to vaccinate. However, the AstraZeneca Vaxzevria COVID-19 vaccine was less favoured because of issues concerning vaccine induced thrombocytopenia. Most individuals stated they were willing to get any COVID-19 vaccine offered:

Selena: For me, I would say, it doesn't affect it as much … If I were sitting there with two different needles in front of me, and one was higher effectiveness than the other, maybe I would choose the higher effectiveness one because they're both there.

Government mandates and policies were acknowledged to impact the decision to vaccinate. However, most participants expressed strong intentions regardless of the messaging and rules:

Tony: I wanted to get the vaccine whether or not there was pressure, but I do think that there is a large pressure put on our different institutions and our government officials pushing us, ‘*You need to get this vaccine. You need to get this vaccine.’* Whether they're right or not, I'm not going to comment on that. Like I already said, the right thing to do is to get the vaccine but I think there definitely is pressure.

There were criticisms related to government websites, the online booking system, and the possibility that public health's actions may have negatively influenced patient-provider trust because of the politicization of the COVID-19 vaccine. Additionally, they felt the policies, mandates, and strong advocacy in Canada led to some pushback and discontent:

Julian: Well, I think my biggest gripe is that we should have acknowledged that public health and healthcare in general we have a finite amount of let's call it social capital with the public. And we expended that very, very quickly on items that honestly was a little bit of a farce. We said ‘*Go home for two weeks. If you're there for two weeks, it will go away. We don't have to worry. This is all we need from you.’* In my lifetime, I've never seen a community [come] together as quickly and as strongly as that. But at the same time, I think we also used a lot of our social capital almost immediately in the pandemic instead of considering the fact that this could be long-term.

## 4. Discussion

We conducted these semi-structured interviews shortly after Canada approved the use of the third COVID-19 vaccine dose for adults (Public Health Agency of Canada, 2022). We applied the TDF and used thematic analysis to generate overarching themes and identify the major drivers towards HCPs’ decision to get vaccinated. Overall, participants in this study reported factors such as evidence-informed decision-making, sense of responsibility, accessibility, normative influences, and policy which influenced their decision to get the COVID-19 and influenza vaccines. The recommendations below are starting points in which to shape future interventions and messaging rather than serving as a one-size-fits-all approach.

Several notable internal, interpersonal, and environmental factors were identified that influence vaccine uptake. Previous research found similar reasons for vaccine uptake among healthcare providers (Halpin & Reid, 2022; Mustapha, Khubchandani, & Biswas, 2021; Yassi et al., 2010). Beliefs about consequences, knowledge, and social influences could be mechanisms of action (MoA) targeted to maintain and strengthen intentions toward these vaccines. Behaviour change techniques that have been demonstrated to bring about change through these MoAs include providing information about health consequences of COVID-19 and influenza, information about social and environmental consequences of getting vaccinated (e.g. benefit to the community), and information about others’ approval (i.e. what other patients and HCPs think about getting vaccinated) (Carey et al., 2019). Messaging can be modified to include some knowledge gaps highlighted in our study, such as emerging data and the efficacy of third doses in the short – and medium-term. Positive reinforcement in the form of incentives have also been demonstrated to improve beliefs about consequences and intentions toward a behaviour (Carey et al., 2019). Therefore, the use of promotional campaigns in combination with BCTs at occupational settings and making vaccines widely accessible free of cost can lead to increased uptake and favourable perceptions toward vaccines as found in our study and in previous research (Burnett et al., 2021; Harris et al., 2011; Yue et al., 2019).

Regarding external factors, the availability of vaccines in combination with organizational policies and government mandates were primary external drivers influencing vaccine uptake among HCPs interviewed in this study. Moreover, many opinions were expressed relating to the larger healthcare infrastructure and government bodies. Participants in our study viewed themselves as being part of these institutions. There were some appeals and criticisms to implementing vaccination requirements for individuals working in healthcare, and similar sentiments have been shared in previous research with varying levels of support by participants and researchers on mandates (Bradfield & Giubilini, 2021; Gagneux-Brunon et al., 2022; Gualano et al., 2021; Ottenberg et al., 2011; Yassi et al., 2010), vaccine passports (Forman et al., 2021), and the ethical rationale for prioritizing welfare over autonomy (Bradfield & Giubilini, 2021; Tilburt et al., 2008). Although some workplaces and regions do not require their employees to receive influenza vaccinations (Gruben, Siemieniuk, & McGeer, 2014), previous research has shown mandated vaccinations to be effective at increasing coverage (Hollmeyer et al., 2013; Van Buynder et al., 2015). Implementing vaccination programmes at a healthcare setting, mandatory or otherwise, may also be cost effective through the reduction of hospital-acquired infections, improved patient outcomes, and reduced worker absenteeism (Chan, 2007; Saxén & Virtanen, 1999; Verelst et al., 2021; Wilde et al., 1999). Condition-of-service vaccination policies such as vaccinate-or-mask and required vaccinations have been shown to increase influenza vaccination coverage (Gruben et al., 2014), but it is not without criticisms (Yassi et al., 2010). Thus, it may be beneficial to have vaccination campaigns seasonally and provide HCPs with sufficient resources and capacity to receive the vaccine as well as deliver a high standard of patient education. As previous research suggests (Gruben et al., 2014; Harris et al., 2011; Hollmeyer et al., 2013; Maurer et al., 2012; Wang, Jing, & Bocchini, 2017; Yassi et al., 2010), the use of multipronged approaches which improve workplace safety culture (i.e. providing a more holistic approach to health and safety information rather than a singular focus on vaccination), increase knowledge to promote evidence-informed decisions, shift away from coercion and punishments, and increase accessibility and availability may improve support for policies, increase long-term vaccine uptake, and result in improved provider-patient health communication and vaccine education.

### 4.1. Strengths and limitations

This was the first study of its kind to combine the topics of influenza and COVID-19 vaccines into a qualitative investigation of HCPs to compare barriers to uptake. We applied the TDF to the study design, interview guide, and analysis which offered several benefits. It provided rich and diverse insights into HCPs perspectives surrounding vaccines, which can be linked directly to evidence-based strategies to increase vaccination coverage as well as speed of uptake. Moreover, the application of this framework allowed several determinants of vaccine uptake to be considered rather than selecting one theoretical perspective to guide this research. The identification of these modifiable determinants of behaviour can allow researchers to bridge the research and practice gap and link these determinants to behaviour change techniques. However, we recognize that using the TDF also has its limitations as it is a rigid framework, may prompt forced responses from participants, and encompasses a broad range of determinants which can lead to challenges in identifying key barriers (Debono et al., 2017; Griffiths et al., 2021; Lawton et al., 2016). We used the TDF more flexibly by having a semi-structured interview guide, using both deductive and inductive analysis, and distilling the findings into key determinants to overcome these limitations.

Multiple professions were included to map broader challenges and concerns regarding these vaccines. As is the case in most qualitative research studies, our findings are context-dependent and not generalizable to a broader population. In fact, much of the discourse centred around COVID-19 vaccines and participants had to be prompted to discuss influenza, which was expected given that the COVID-19 pandemic was more pressing. Additional COVID-19 vaccine doses, vaccine formulations, and barriers to vaccine uptake may change with time. Furthermore, many professions were not captured in this study and others, such as dietitians and pharmacists, were only covered to a small extent. HCPs also make up a variety of individuals from diverse backgrounds including age, education, ethnicity, culture, and socioeconomic status. We did not find any profession-specific differences among the individuals interviewed except under the environment, organizational context, and resources domain. However, it is also unlikely we reached data saturation among these groups. Lastly, most participants in this study were fully vaccinated and their barriers to uptake may be different than those who are hesitant or unvaccinated. Due to the lack of voices from unvaccinated and more vaccine-hesitant providers in our study, we acknowledge this study may not provide a complete list of notable barriers all Canadian HCPs face when considering COVID-19 and influenza vaccinations for themselves. Therefore, we were unable to comment on these aspects beyond the participants’ experiences and anecdotes shared across interviews.

### 4.2. Future research

To further explore providers’ beliefs, the methodology used in this study can be applied to groups not captured in this study such as vaccine-hesitant providers, unvaccinated providers, and other professions (e.g. general practitioners, social workers). Additionally, we encourage researchers to collect detailed demographic factors such as age, ethnicity, and location. This may help shed light on whether similar barriers are being faced across professions, age groups, geographic regions, ethnicities, and vaccine-hesitant individuals. Identification of any disparities in barriers across HCPs may allow future interventions to be tailored to certain groups or provide justification for upstream changes such as modifying the educational curriculum of select healthcare fields. This study can also benefit from a replication in the future as vaccine sentiments, risk perceptions, policies, boosters, and additional COVID-19 vaccines approved for use may change with time. Quantitative studies in this area could measure some of the variables identified in this research and assess whether they predict intentions to get vaccinated.

## 5. Conclusions

This study used a theoretical framework to identify barriers and facilitators toward COVID-19 and influenza vaccine uptake among Canadian HCPs and trainees. Overall, we found participants made evidence-informed health decisions, felt a strong duty to protect themselves and patients, but some questioned vaccine mandates, and were apprehensive of COVID-19 vaccines due to the lack of long follow up data and potential side effects. Future avenues for messaging and interventions to overcome barriers include delivering vaccination campaigns to increase knowledge of the health consequences of these pathogens, providing a greater level of scientific information to HCPs to increase capacity of HCPs, maximizing vaccine access at occupational settings, and supplementing vaccine mandates with education to increase support for these policies. Future research is warranted to further explore barriers to vaccine uptake among other healthcare professions and unvaccinated providers not covered in this study.

## Data Availability

Participants did not provide consent for their data to be made publicly available. However, we will supply the relevant code files in the appendix. Output files derived from the analyses can be provided upon request. The authors would like to thank the participants who generously gave their time to share their experiences for this study. We would also like to thank the Canadian Public Health Association and Melissa Wong for their in-kind assistance with participant recruitment.

## Appendix

**Semi-structured interview guide for healthcare providers on barriers and facilitators of COVID-19 and influenza vaccine uptake.**

*We will begin with some general questions.*

1. What is your primary occupation?
2. How familiar are you with COVID-19 and influenza vaccines?
3. Can you tell me what your general views are on these vaccines?
4. Did you get the COVID-19 vaccines? Do you normally get vaccinated during the flu season?
5. For you personally, are there any factors which make it easy or difficult in getting vaccinated?

*We have some questions about more specific factors that we think might play a role in the extent to which recommendations are followed*.

1. Knowledge
  - Are there any gaps in what you know about COVID-19 and influenza vaccines?
  - What sources do you use to inform yourself on vaccines?
2. Product
  - Does the reputation or effectiveness of a vaccine impact whether or not you choose to get it?
3. Social/professional role and identity
  - Do you feel it is your responsibility to get vaccinated?
4. Capabilities and skills
  - How easy or difficult was it for you to receive the COVID-19 vaccines? How does that compare with the flu shot?
5. Beliefs about consequences
  - In your opinion, do the benefits of following these recommendations outweigh the costs? Can you compare between COVID-19 and influenza vaccines?
6. Motivation and goals
  - For you personally, are there any incentives for getting vaccinated? Is there a difference between COVID-19 and influenza vaccines?
7. Emotion
  - Do your feelings or mood affect whether or not you choose to receive a vaccine?
8. Memory, attention, and decision processes; behavioural regulation
  - Are there things that help to prompt you to get vaccinated?
  - **[Influenza only]** Do you usually remember to get the influenza vaccine?
9. Environmental context and resources
  - Does the accessibility of a vaccine impact you in wanting to get or not to get vaccine?
10. Social and organizational influences
  - Do people you work with get vaccinated? What about your friends and family members?
  - Do your feel your workplace affects whether or not you get vaccinated?
  - If someone you know got the flu or COVID-19, would that affect whether or not you get the vaccine for yourself? Or would it not affect your decision?
11. Societal and cultural influence
  - Have you ever felt pressured from your community, culture, or religion to get the COVID-19 or influenza vaccine?
12. Government/organization
  - Do you feel the government or public health affects whether or not you get vaccinated?
13. Any other factors that you think might be important that we haven’t covered?

**Codebook for the qualitative study exploring healthcare providers’ barriers and facilitators toward the uptake of COVID-19 and influenza vaccines.**

**Table:**

Domain or Category	Comments
Knowledge	Preferred channels; influence of mis/disinformation
Professional role and identity	Set of behaviours and personal qualities expressed in a social or work setting
Beliefs about capabilities	Confidence to carry out a set of behaviours
Beliefs about consequences	Exposure to or experience with COVID, including natural immunity; severity and susceptibility related to their individual factors
Product	Reputation, brand, and effectiveness; thoughts on boosters including within the context of variants; regulatory processes related to vaccine approval including pace of development; side effects, risk of vaccine, safety concerns; confidence in benefits; mRNA technology
Reinforcement	Increasing the probability of a response by demonstrating some stimulus-response relationship
Goals	Mental representations of outcomes or end states that an individual wants to achieve
Intentions	Conscious decision to perform a behaviour or a resolve to act in a certain way
Memory, attention, and decision processes	Retain information or focus selectively on the environment and choose between alternatives
Emotion	Complex affective factors which is experiential, behavioural, and physiological by which vaccine-related thoughts or events are processed
Behavioural regulation	Anything aimed at managing or changing objectively observed or measured actions
Environmental context and resources	Availability and ease of access on site
Social influence	Friends, family, colleagues, classmates, and significant other
Organizational factors	Goes beyond colleagues, and mentions work policies, provisions, etc.; pressure from workplace or educational institution
Cultural and societal factors	People from immigrant families, cultural beliefs, and familial norms
Policies	Impact of mandates, vaccine passports, did other mandates like travel play a role in decision?
Government and public health	Needs assessment on messaging and communication; preferred spokespersons; depth of information on efficacy and safety
Trust	Patient-provider trust; government-provider trust

**Themes and domains accompanied with additional exemplar quotes.**

**Table:**

Theme	Domain or construct	Illustrative quote(s) on facilitators and barriers to vaccine uptake
Making informed health decisions with an added responsibility to protect oneself and patients	Knowledge	Rico: In the peak of when the vaccines were coming out, I was constantly browsing PubMed, NCBI and looking for papers with actual credible authors. Making sure that they've had past publications and trying to see whether or not I could actually validate the studies based on the way they were carried out. Selena: I actually love Twitter as well. There are a lot of good people on Twitter that – and not to say that I take what I read on Twitter as fact. I definitely like reading dialogues on Twitter. I think those have been really informative to get both sides. Real life experiences. Victoria: … misinformation, it's been a thing to manage, and I do wish we would have been given resources as nurses on how to deal with misinformation, how to deal with like commonly asked questions, and how to explain the vaccines [*sic*] mechanism action, and the easy way how to explain the reason you get boosters, or the reason you need two doses. The reasons why there's different vaccines in the country like Moderna, Pfizer mostly. I think we aren't being provided with enough resources … [It] would have been nice to see more action on misinformation because morally and personally it's just really discouraging to see those types of things. Jade: There are a lot of barriers that can prevent patients from being able to get the vaccine and one of the barriers I think is misinformation surrounding the vaccine and how difficult it is to get reliable information about the vaccine. Lindsay: It was the 60s and 70s where these influential vaccines were initiated, and we all got them as kids or you know, great majority of kids get them, and I have no problem with that. So, knowing that, you know, those six or seven we all get as kids eradicated horrible viruses gives me a bit more comfort in trusting science and getting whatever comes our way now.
	Professional role and identity	Katherine: As a future health care professional, I feel like I need to model that. I feel like people would judge me if I was providing this care to these people and I was not vaccinated myself. Wendy: Obviously, I'm pro-vaccine. I'm a medical student. Jade: There's definitely a social responsibility and a professional responsibility to get vaccinated, but I wouldn't say that that really influenced my decision because my views are so aligned with those responsibilities. They exist, but I don't think they influenced me too much.
	Beliefs about consequences	Sayyid: I think by getting vaccinated for both influenza and COVID, I hope that I'm doing my best to protect these people [even] if I don't know them, but especially as a healthcare professional, you know? When I work in a hospital or clinic, I see these people. We're in the same room … breathing … we're in the same space. And so I think it's really important to consider these people and say, ‘*Yes, I'm protecting myself, but the bigger reason is I'm protecting the people around me,’* which is so so important. Because we have to look out for each other I think as a community and the healthcare system.
	Emotions	Alejandra: … I was super happy after getting all my vaccines. I just felt really positive. Tony: I think that when you want to decide to get a vaccination, that is a long-term decision that you are making about your own body. You're putting something that can have effects on you, and you need to study that, and I think that your decision shouldn't be related to your mood. That's just my own opinion. Sayyid: I try not to let emotions get in the way of logical thought. Claire: I do feel sketchy about what I gave to my body. I do and I did feel that way, even receiving the vaccines and the long-term effects of it because as much as they say *Oh, they have been studied.* There's no possible way the long-term effects have been studied with it being around for such a short period of time.
A pro-vaccine social network, widespread accessibility, and pursuing a sense of normalcy	Social influences	Xue: My husband I guess was the biggest influencer on me. Noelle: I remember speaking to a couple of my friends were nurses and they sent me links to study on it. Sayyid: My roommate (a medical student, confirmed earlier in the interview) actually does a lot of research on COVID – not for work, but because he finds it fascinating. When we have dinner or we meet up during the day, he tells me something he learned today from the vaccine, and so that helped improve my knowledge.
	Societal and cultural norms	Claire: I feel if I wasn't vaccinated, I would feel like a black sheep. Like I said, the two colleagues that I do know that were unvaccinated struggled heavily with mental health and feeling ostracized and people looked at them differently. Elisabeth: I just want this to be over. Like, I want to stop opening up the news everyday it's like ‘*COVID article, COVID article, COVID this, COVID that.’* The whole reason that the majority of people get vaccinated, like, let's get most people vaccinated, is so that we can stop talking about this, you know? So that it can become like the flu where it's not a big deal. Every year, we just get our shot and move on and stop talking about [it].
	Environment, organizational context, and resources	Jilo: For the first one, it was a little bit more stressful and frustrating to deal with that provincial website. Wendy: I was one of the earlier ones who got – who was eligible for the first dose – and through my school it was pretty easy to book. So I mean, they did it at my school. It was right across the street from me, so it was pretty easy. Yeah, I had no problems booking it. Wendy: Now, I'm more inclined to get the flu shot because I'd be around a lot more people and people who are immunocompromised especially in the hospital. So yeah, I’d say it definitely changed my perspective a little bit. Julian: I'm sure you heard about, you know, those nurses and other physicians that were no longer allowed to come into hospitals and whatnot once policies changed for COVID-19. And same thing with the flu. It's also mandatory for us to go and do that. Ellen: When we were first getting it and there was no online booking system in place for the first three hours of the day, our phones would ring nonstop, and we had five lines and every single line would be ringing with people trying to call and make an appointment and we were never in control of how many vaccines we got. It was the Ministry [of Health] sent us what they felt like we should have.
Seeking a more nuanced, respectful, and calculated approach to vaccine communication and policy implementation	Skepticism of pharmaceutical companies	Ellen: … we kind of knew it wasn't effective against the current (Omicron) variant of COVID-19. Personally, I didn't really see a point in getting a third dose of something that wasn't really doing much. But I was also influenced by the fact that I already had COVID a year ago. So between having the virus and then also getting the vaccine, that was the only time I really questioned like, *What's the point of doing this third dose if it's minimally effective?* Madeline: Whenever people ask me questions like, *Oh, how do you know this is safe?* I always give the example [of] things we put in our body that we absolutely have no idea, and we still do it. One example I always give is, I used to be on birth control, and I think it's still not very well understood and something I had gone off later on down the road just because of how much it was affecting me. Xue: I would rather not get a vaccine unless I absolutely had to. And I feel that way about everything, like medication in general. So unless it was absolutely necessary, or I found it absolutely necessary, I would rather avoid putting something like that into my body.
	Government and public health	Selena: It's required for school, it's required for travel, and things like that might be looked at as incentives. Personally, those were just bonuses for me. I think I would have, whether it was required for anything or not, I would have gotten it. Claire: Communication was incredibly poor throughout it all. It's so great … back and forth, back and forth. Going into work and new rules each day, each week. And then us having to relate this back to our patients and families. It was a very great time of trying to figure out what is going on and I do appreciate that it was like that for everybody. This isn't just a single personal experience. But could it have been better handled? Yes. Do I know how? No. Elisabeth: Every once in a while, you get someone still coming in just now for their first [COVID-19 vaccine dose] and they're angry like they're not happy about it. And I've had people sit in that counselling room with me just complaining. Like, while I sit there, just angry about being supposedly forced into getting the vaccine and for whatever reason the government or the workplace or to go to restaurants, and they're not happy about it. Sayyid: [Ottawa Public Health's] website does a really great job of disseminating [*sic*] myths about the vaccine and COVID. I follow them on Twitter, and they do a fantastic job of kind of just like, if they hear something that was misinformation, they post on Twitter and they explain why it's factually incorrect. Or they'll do something and then amplify [that message] and say, *‘Oh, look, we have COVID vaccines available here. Here are the ones you have available. Here's the evidence behind it.’*

## References

[CIT0001] Andre FE, Booy R, Bock HL, Clemens J, Datta SK, John TJ, ... & Schmitt HJ. (2008) Vaccination greatly reduces disease, disability, death and inequity worldwide. *Bulletin of the World Health Organization* *86*(2): 140. doi:10.2471/BLT.07.040089. PMC2647387 18297169

[CIT0002] Bradfield, O. M., & Giubilini, A. (2021). Spoonful of honey or a gallon of vinegar? A conditional COVID-19 vaccination policy for front-line healthcare workers. *Journal of Medical Ethics*, *47*(7), 467–472. doi:10.1136/MEDETHICS-2020-107175 PMC8257552 33975928

[CIT0003] Bradshaw, C., Atkinson, S., & Doody, O. (2017). Employing a qualitative description approach in health care research. *Global Qualitative Nursing Research*, *4*. doi:10.1177/2333393617742282 PMC5703087 29204457

[CIT0004] Braun, V., & Clarke, V. (2006). Using thematic analysis in psychology. *Qualitative Research in Psychology*, *3*(2), 77–101.

[CIT0005] Burnett, R. J., Dramowski, A., Amponsah-Dacosta, E., et al. (2021). Increasing hepatitis B vaccination coverage of healthcare workers - global lessons for South Africa. *Current Opinion in Immunology*, *71*, 6–12. doi:10.1016/J.COI.2021.03.010 33819774

[CIT0006] Cane, J., O’Connor, D., & Michie, S. (2012). Validation of the theoretical domains framework for use in behaviour change and implementation research. *Implementation Science*, *7*(1), 1–17. doi:10.1186/1748-5908-7-37/TABLES/3 PMC3483008 22530986

[CIT0007] Carey, R. N., Connell, L. E., Johnston, M., et al. (2019). Behavior change techniques and their mechanisms of action: A synthesis of links described in published intervention literature. *Annals of Behavioral Medicine*, *53*(8), 693–707. doi:10.1093/ABM/KAY078 30304386 PMC6636886

[CIT0008] Chan, S. S. W. (2007). Does vaccinating ED health care workers against influenza reduce sickness absenteeism? *The American Journal of Emergency Medicine*, *25*(7), 808–811. doi:10.1016/J.AJEM.2007.02.002 17870487

[CIT0009] Clark, S. J., Cowan, A. E., & Wortley, P. M. (2009). Influenza vaccination attitudes and practices among US registered nurses. *American Journal of Infection Control*, *37*(7), 551–556. doi:10.1016/j.ajic.2009.02.012 19556035

[CIT0010] Debono, D., Taylor, N., Lipworth, W., et al. (2017). Applying the theoretical domains framework to identify barriers and targeted interventions to enhance nurses’ use of electronic medication management systems in two Australian hospitals. *Implementation Science*, *12*(1), 1–13. doi:10.1186/S13012-017-0572-1/TABLES/5 28347319 PMC5368903

[CIT0011] Di Giuseppe, G., Pelullo, C. P., Paolantonio, A., et al. (2021). Healthcare workers’ willingness to receive influenza vaccination in the context of the COVID-19 pandemic: A survey in southern Italy. *Vaccines*, *9*(7), 766. doi:10.3390/VACCINES9070766 34358182 PMC8310353

[CIT0012] Dubé, E., Gagnon, D., Kiely, M., et al. (2014). Seasonal influenza vaccination uptake in Quebec, Canada, 2 years after the influenza A (H1N1) pandemic. *American Journal of Infection Control*, *42*(5), e55–e59. doi:10.1016/J.AJIC.2014.01.006 24773805

[CIT0013] Dubé, E., Laberge, C., Guay, M., et al. (2013). Vaccine hesitancy: An overview. *Human Vaccines and Immunotherapeutics*. doi:10.4161/hv.24657 PMC3906279 23584253

[CIT0014] Forman, R., Shah, S., Jeurissen, P., et al. (2021). COVID-19 vaccine challenges: What have we learned so far and what remains to be done? *Health Policy*, *125*(5), 553–567. doi:10.1016/J.HEALTHPOL.2021.03.013 33820678 PMC7997052

[CIT0015] Gagneux-Brunon, A., Botelho-Nevers, E., Bonneton, M., et al. (2022). Public opinion on a mandatory COVID-19 vaccination policy in France: A cross-sectional survey. *Clinical Microbiology and Infection*, *28*(3), 433–439. doi:10.1016/J.CMI.2021.10.016 34774756 PMC8912915

[CIT0016] Gagneux-Brunon, A., Detoc, M., Bruel, S., et al. (2021). Intention to get vaccinations against COVID-19 in French healthcare workers during the first pandemic wave: A cross-sectional survey. *Journal of Hospital Infection*, *108*, 168–173. doi:10.1016/J.JHIN.2020.11.020 33259883 PMC7699157

[CIT0017] Gallant, A. J., Flowers, P., Deakin, K., et al. (2021). Barriers and enablers to influenza vaccination uptake in adults with chronic respiratory conditions: Applying the behaviour change wheel to specify multi-levelled tailored intervention content. *Psychology & Health*. doi:10.1080/08870446.2021.1957104 PMC9970185 34328044

[CIT0018] Genovese, C., Picerno, I. A. M., Trimarchi, G., et al. (2019). Vaccination coverage in healthcare workers: A multicenter cross-sectional study in Italy. *Journal of Preventive Medicine and Hygiene*, *60*(1), E12–E17. doi:10.15167/2421-4248/JPMH2019.60.1.1097 31041405 PMC6477557

[CIT0019] Green, J., & Thorogood, N. (2018). *Qualitative methods for health research* (D Silvermaned.). 4th ed. London: SAGE Publications Ltd. Retrieved April 22, 2022, from https://us.sagepub.com/en-us/nam/qualitative-methods-for-health-research/book254905

[CIT0020] Green-Mckenzie, J., Shofer, F. S., Momplaisir, F., et al. (2021). Factors associated with COVID-19 vaccine receipt by health care personnel at a major academic hospital during the first months of vaccine availability. *JAMA Network Open*, *4*(12), e2136582–e2136582. doi:10.1001/JAMANETWORKOPEN.2021.36582 34851399 PMC8637254

[CIT0021] Griffith, J., Marani, H., & Monkman, H. (2021). COVID-19 vaccine hesitancy in Canada: Content analysis of tweets using the theoretical domains framework. *Journal of Medical Internet Research*, *23*(4), e26874. doi:10.2196/26874 33769946 PMC8045776

[CIT0022] Griffiths, S. E., Naughton, F., & Brown, K. E. (2021). Accessing specialist support to stop smoking in pregnancy: A qualitative study exploring engagement with UK-based stop smoking services. *British Journal of Health Psychology*, doi:10.1111/BJHP.12574 PMC9542141 34852182

[CIT0023] Grochowska, M., Ratajczak, A., Zdunek, G., et al. (2021). A comparison of the level of acceptance and hesitancy towards the influenza vaccine and the forthcoming COVID-19 vaccine in the medical community. *Vaccines*, *9*(5), 475. doi:10.3390/VACCINES9050475 34066790 PMC8150871

[CIT0024] Gruben, V., Siemieniuk, R. A., & McGeer, A. (2014). Health care workers, mandatory influenza vaccination policies and the law. *Canadian Medical Association Journal*, *186*(14), 1076. doi:10.1503/CMAJ.140035 24863919 PMC4188651

[CIT0025] Gualano, M. R., Corradi, A., Voglino, G., et al. (2021). Healthcare workers’ (HCWs) attitudes towards mandatory influenza vaccination: A systematic review and meta-analysis. *Vaccine*, *39*(6), 901–914. doi:10.1016/J.VACCINE.2020.12.061 33451776

[CIT0026] Halpin, C., & Reid, B. (2022). Attitudes and beliefs of healthcare workers about influenza vaccination. *Nursing Older People* 34Halpin C(2). doi:10.7748/NOP.2019.E1154. 31468782

[CIT0027] Harris, K., Maurer, J., Black, C., et al. (2011). Workplace efforts to promote influenza vaccination among healthcare personnel and their association with uptake during the 2009 pandemic influenza A (H1N1). *Vaccine*, *29*(16), 2978–2985. doi:10.1016/J.VACCINE.2011.01.112 21334387

[CIT0028] Hollmeyer, H., Hayden, F., Mounts, A., et al. (2013). Review: Interventions to increase influenza vaccination among healthcare workers in hospitals. *Influenza and Other Respiratory Viruses*, *7*(4), 604–621. doi:10.1111/IRV.12002 22984794 PMC5781006

[CIT0029] Hollmeyer, H. G., Hayden, F., Poland, G., et al. (2009). Influenza vaccination of health care workers in hospitals - A review of studies on attitudes and predictors. *Vaccine*, doi:10.1016/j.vaccine.2009.03.056 19467744

[CIT0030] Kim, M. H., Son, N. H., Park, Y. S., et al. (2021). Effect of a hospital-wide campaign on COVID-19 vaccination uptake among healthcare workers in the context of raised concerns for life-threatening side effects. *PLOS ONE*, *16*(10), e0258236. doi:10.1371/JOURNAL.PONE.0258236 34597333 PMC8486118

[CIT0031] Kirk, J. W., Sivertsen, D. M., Petersen, J., et al. (2016). Barriers and facilitators for implementing a new screening tool in an emergency department: A qualitative study applying the theoretical domains framework. *Journal of Clinical Nursing*, *25*(19–20), 2786–2797. doi:10.1111/jocn.13275 27273150

[CIT0032] Kwok, K. O., Li, K. K., Wei, W. I., et al. (2021). Influenza vaccine uptake, COVID-19 vaccination intention and vaccine hesitancy among nurses: A survey. *International Journal of Nursing Studies*, *114*, 103854. doi:10.1016/j.ijnurstu.2020.103854 33326864 PMC7831770

[CIT0033] Lam, P. P., Chambers, L. W., Pierrynowski MacDougall, D. M., et al. (2010). Seasonal influenza vaccination campaigns for health care personnel: Systematic review. *Canadian Medical Association Journal*, *182*(12), E542–E548. doi:10.1503/CMAJ.091304 20643836 PMC2934816

[CIT0034] Latkin, C. A., Dayton, L., Miller, J. R., et al. (2021). Behavioral and attitudinal correlates of trusted sources of COVID-19 vaccine information in the US. *Behavioral Sciences*, *11*(4), 56. doi:10.3390/bs11040056 33924118 PMC8074305

[CIT0035] Lauring, A. S., Tenforde, M. W., Chappell, J. D., et al. (2022). Clinical severity of, and effectiveness of mRNA vaccines against, COVID-19 from omicron, delta, and alpha SARS-CoV-2 variants in the United States: Prospective observational study. *BMJ*, *376*. doi:10.1136/BMJ-2021-069761 PMC8905308 35264324

[CIT0036] Lawton, R., Heyhoe, J., Louch, G., et al. (2016). Using the theoretical domains framework (TDF) to understand adherence to multiple evidence-based indicators in primary care: A qualitative study. *Implementation Science*, *11*(1), 1–16. doi:10.1186/S13012-016-0479-2/TABLES/10 27502590 PMC4977705

[CIT0037] Madill, A., Jordan, A., & Shirley, C. (2000). Objectivity and reliability in qualitative analysis: Realist, contextualist and radical constructionist epistemologies. *British Journal of Psychology*, *91*(1), 1–20. doi:10.1348/000712600161646 10717768

[CIT0038] Maltezou, H. C., Theodoridou, K., Ledda, C., et al. (2019). Vaccination of healthcare workers: Is mandatory vaccination needed? *Expert Review of Vaccines*, *18*(1), 5–13. doi:10.1080/14760584.2019.1552141 30501454

[CIT0039] Maurer, J., Harris, K. M., Black, C. L., et al. (2012). Support for seasonal influenza vaccination requirements among US healthcare personnel. *Infection Control and Hospital Epidemiology*, *33*(3), 213–221. doi:10.1086/664056 22314055

[CIT0040] Mustapha, T., Khubchandani, J., & Biswas, N. (2021). COVID-19 vaccination hesitancy in students and trainees of healthcare professions: A global assessment and call for action. *Brain, Behavior, & Immunity - Health*, *16*, 100289. doi:10.1016/J.BBIH.2021.100289 PMC8241692 34226892

[CIT0041] O’Brien, B. C., Harris, I. B., Beckman, T. J., et al. (2014). Standards for reporting qualitative research: A synthesis of recommendations. *Academic Medicine*, *89*(9), 1245–1251. doi:10.1097/ACM.0000000000000388 24979285

[CIT0042] Ogilvie, G. S., Gordon, S., Smith, L. W., et al. (2021). Intention to receive a COVID-19 vaccine: Results from a population-based survey in Canada. *BMC Public Health*, *21*(1), 1–14. doi:10.1186/s12889-021-11098-9 34051770 PMC8164402

[CIT0043] Ottenberg, A. L., Wu, J. T., Poland, G. A., et al. (2011). Vaccinating health care workers against influenza: The ethical and legal rationale for a mandate. *American Journal of Public Health*, *101*(2), 212. doi:10.2105/AJPH.2009.190751 21228284 PMC3020194

[CIT0044] Pope, C., & Mays, N. (1995). Qualitative research: Reaching the parts other methods cannot reach: An introduction to qualitative methods in health and health services research. *BMJ*, *311*, 42–45. doi:10.1136/BMJ.311.6996.42 7613329 PMC2550091

[CIT0045] Public Health Agency of Canada. (2022). An Advisory Committee Statement (ACS) National Advisory Committee on Immunization (NACI) Initial guidance on a second booster dose of COVID-19 vaccines in Canada. 5 April. Retrieved April 19, 2022, from https://www.canada.ca/content/dam/phac-aspc/documents/services/immunization/national-advisory-committee-on-immunization-naci/naci-guidance-second-booster-dose-covid-19-vaccines.pdf

[CIT0046] Rodrigues, C. M. C., & Plotkin, S. A. (2020). Impact of vaccines; health, economic and social perspectives. *Frontiers in Microbiology*, *11*, 1526. doi:10.3389/FMICB.2020.01526/BIBTEX 32760367 PMC7371956

[CIT0047] Sallam, M. (2021). COVID-19 vaccine hesitancy worldwide: A concise systematic review of vaccine acceptance rates. *Vaccines*, *9*(2), 1–15. doi:10.3390/vaccines9020160 PMC7920465 33669441

[CIT0048] Saxén, H., & Virtanen, M. (1999). Randomized, placebo-controlled double blind study on the efficacy of influenza immunization on absenteeism of health care workers. *The Pediatric Infectious Disease Journal*, *18*(9), 779–783. doi:10.1097/00006454-199909000-00007 10493337

[CIT0049] Schumacher, S., Salmanton-García, J., Cornely, O. A., et al. (2021). Increasing influenza vaccination coverage in healthcare workers: A review on campaign strategies and their effect. *Infection*, *49*(3), 387–399. doi:10.1007/S15010-020-01555-9/FIGURES/2 33284427 PMC7720031

[CIT0050] Shekhar, R., Sheikh, A. B., Upadhyay, S., et al. (2021). COVID-19 vaccine acceptance among health care workers in the United States. *Vaccines*, *9*(2), 1–18. doi:10.3390/vaccines9020119 PMC7913135 33546165

[CIT0051] Silva, J., Bratberg, J., & Lemay, V. (2021). COVID-19 and influenza vaccine hesitancy among college students. *Journal of the American Pharmacists Association*, *61*(6), 709–714. doi:10.1016/J.JAPH.2021.05.009 34092517 PMC8139529

[CIT0052] Statistics Canada. (2019). Primary health care providers, 2017. Retrieved May 19, 2022, from https://www150.statcan.gc.ca/n1/pub/82-625-x/2019001/article/00001-eng.htm

[CIT0053] Tilburt, J. C., Mueller, P. S., Ottenberg, A. L., et al. (2008). Facing the challenges of influenza in healthcare settings: The ethical rationale for mandatory seasonal influenza vaccination and its implications for future pandemics. *Vaccine*, *26*(SUPPL. 4), D27–D30. doi:10.1016/J.VACCINE.2008.07.068 19230155

[CIT0054] To, K. W., Lai, A., Lee, K. C. K., et al. (2016). Increasing the coverage of influenza vaccination in healthcare workers: Review of challenges and solutions. *Journal of Hospital Infection*, *94*(2), 133–142. doi:10.1016/J.JHIN.2016.07.003 27546456

[CIT0055] Van Buynder, P. G., Konrad, S., Kersteins, F., et al. (2015). Healthcare worker influenza immunization vaccinate or mask policy: Strategies for cost effective implementation and subsequent reductions in staff absenteeism due to illness. *Vaccine*, *33*(13), 1625–1628. doi:10.1016/J.VACCINE.2015.01.048 25678243

[CIT0056] Verelst, F., Beutels, P., Hens, N., et al. (2021). Workplace influenza vaccination to reduce employee absenteeism: An economic analysis from the employers’ perspective. *Vaccine*, *39*(14), 2005–2015. doi:10.1016/J.VACCINE.2021.02.020 33632564

[CIT0057] Wang, T. L., Jing, L., & Bocchini, J. A. (2017). Mandatory influenza vaccination for all healthcare personnel: A review on justification, implementation and effectiveness. *Current Opinion in Pediatrics*, *29*(5), 606–615. doi:10.1097/MOP.0000000000000527 28700416

[CIT0058] Wilde, J. A., McMillan, J. A., Serwint, J., et al. (1999). Effectiveness of influenza vaccine in health care professionals: A randomized trial. *JAMA*, *281*(10), 908–913. doi:10.1001/JAMA.281.10.908 10078487

[CIT0059] Williams, L., Gallant, A. J., Rasmussen, S., et al. (2020). Towards intervention development to increase the uptake of COVID-19 vaccination among those at high risk: Outlining evidence-based and theoretically informed future intervention content. *British Journal of Health Psychology*, *25*(4), 1039–1054. doi:10.1111/BJHP.12468 32889759

[CIT0060] Yassi, A., Lockhart, K., Buxton, J. A., et al. (2010). Vaccination of health care workers for influenza: Promote safety culture, not coercion. *Canadian Journal of Public Health*, *101*(Suppl 1), S41–S45. doi:10.1007/BF03403845 20629446 PMC6974253

[CIT0061] Yue, X., Black, C., Ball, S., et al. (2019). Workplace interventions and vaccination-related attitudes associated with influenza vaccination coverage among healthcare personnel working in long-term care facilities, 2015‒2016 influenza season. *Journal of the American Medical Directors Association*, *20*(6), 718–724. doi:10.1016/J.JAMDA.2018.11.029 30711462 PMC6538419

[CIT0062] Zimmerman, R. K., Bradford, B. J., Janosky, J. E., et al. (1997). Barriers to measles and pertussis immunization: The knowledge and attitudes of Pennsylvania primary care physicians. *American Journal of Preventive Medicine*, *13*(2), 89–97. doi:10.1016/s0749-3797(18)30204-6 9088444

